# Variant biomarker discovery using mass spectrometry-based proteogenomics

**DOI:** 10.3389/fragi.2023.1191993

**Published:** 2023-04-24

**Authors:** Luke Reilly, Sahba Seddighi, Andrew B. Singleton, Mark R. Cookson, Michael E. Ward, Yue A. Qi

**Affiliations:** ^1^ Center for Alzheimer’s and Related Dementias (CARD), National Institute on Aging and National Institute of Neurological Disorders and Stroke, National Institutes of Health, Bethesda, MD, United States; ^2^ National Institute of Neurological Disorders and Stroke, National Institutes of Health, Bethesda, MD, United States; ^3^ Laboratory of Neurogenetics, National Institute on Aging, National Institutes of Health, Bethesda, MD, United States

**Keywords:** biomarker, proteogenomics, aging, neurodegenerative, cancers

## Abstract

Genomic diversity plays critical roles in risk of disease pathogenesis and diagnosis. While genomic variants—including single nucleotide variants, frameshift variants, and mis-splicing isoforms—are commonly detected at the DNA or RNA level, their translated variant protein or polypeptide products are ultimately the functional units of the associated disease. These products are often released in biofluids and could be leveraged for clinical diagnosis and patient stratification. Recent emergence of integrated analysis of genomics with mass spectrometry-based proteomics for biomarker discovery, also known as proteogenomics, have significantly advanced the understanding disease risk variants, precise medicine, and biomarker discovery. In this review, we discuss variant proteins in the context of cancers and neurodegenerative diseases, outline current and emerging proteogenomic approaches for biomarker discovery, and provide a comprehensive proteogenomic strategy for detection of putative biomarker candidates in human biospecimens. This strategy can be implemented for proteogenomic studies in any field of enquiry. Our review timely addresses the need of biomarkers for aging related diseases.

## 1 Application of proteogenomics in biomarker discovery

A biomarker is defined as a biological characteristic that indicates clinically relevant endpoints and outcomes for disease diagnosis, stratification, and/or prognosis (Aronson and Ferner, 2017). To date, biomarkers have been primarily used for early-stage diagnosis, when therapeutic interventions are most effective. Beyond diagnostic applications, biomarkers can also serve as drug targets and proxies of response to treatment. The use of genetic loci as predictive biomarkers has seen a significant advance in recent years, in part due to their high reproducibly and cost-effectiveness which has come with next-generation sequencing (NGS) technology (Schwarze et al., 2018). Disease-based genetics often identifies risk variants associated with diseases, but alone does not provide information on expression at the transcript or protein level. Transcriptome variant markers–such as point mutations, fusion products, and splicing–provide relatively high specificity and sensitivity (Fehse et al., 2000; Janik et al., 2021; Monti et al., 2022). Moreover, while transcriptomics has been widely applied to tissue samples, its application to biofluids is more challenging due to the low quality, quantity, and specificity of RNAs that are recovered from biofluids. The detection of *de novo* protein biomarkers via antibody and mass spectrometry (MS)-based strategies represents a promising solution (Borrebaeck and Wingren, 2009; Zhou et al., 2017). Although immunoassay-based approaches can analyze several proteins at once, they are limited by the availability of suitable antibodies, while MS is generally “hypothesis-free” and high throughput.

Historically, the fields of genomics and proteomics have evolved independently. “Proteogenomics” was first referred to as the application of MS-based proteomics to complement existing genome annotations (Jaffe et al., 2004). The applications have since become much broader, now encompassing post-translational modifications (PTMs) and integrative modeling of multi-omics data with the advent of robust computational tools (Ruggles et al., 2017). Early proteogenomic applications consisted of evaluating parental proteins and their product peptides to identify and validate informatically predicted open reading frames (ORFs), detect *de novo* variants, and reveal PTMs. Now, bioinformatics pipelines allow researchers to combine both genomic and proteomic data in their analyses, making so-called “integrated proteogenomics analyses,” more approachable (Ang et al., 2019).

**FIGURE 1:**
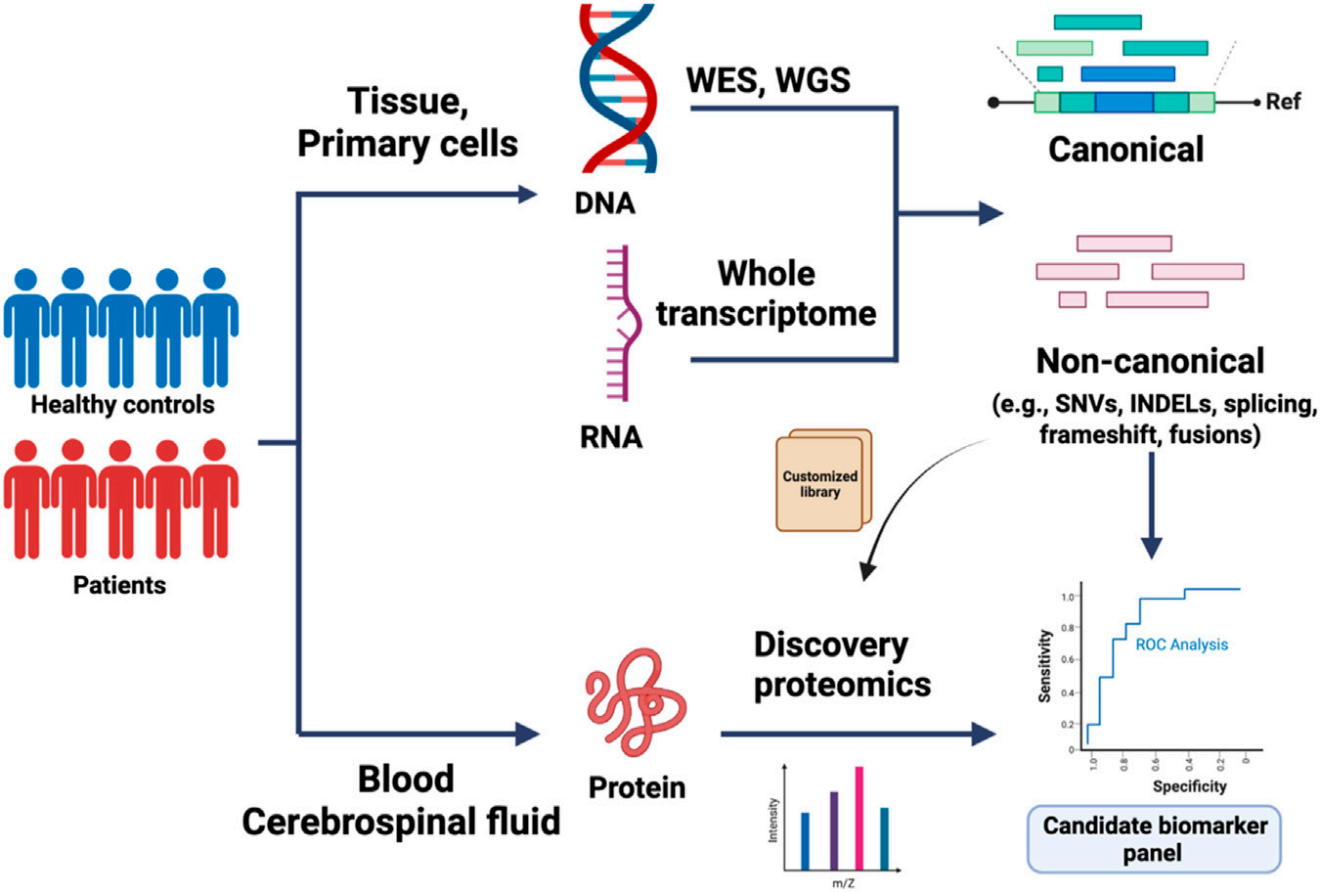
Application of proteogenomics in biomarker discovery. Whole-exome sequencing (WES), whole genome sequencing (WGS), and total RNA sequencing of control and patient-derived samples are used to identify canonical, or *de novo* reads matching to single nucleotide variants (SNVs), short insertions/deletions (indels), mis-splicing, or fusion transcripts. The variant coordinates are integrated within the normal protein sequence to build a custom peptide library. In parallel, discovery proteomics of biofluid samples can be used to build a proteomics database, which can be mined for sequences of interest using the custom-built peptide library. The candidate biomarkers are further validated in large-cohort studies.

In traditional database search strategies for discovery proteomics, experimental protein identification is predicated on the alignment of experimental mass spectra with reference proteome databases, such as the universal Protein Resource (UniProt) and NCBI Reference Sequence Database (Refseq) (Consortium, 2015; O'Leary et al., 2016). With this approach, protein findings are limited to existing sequences within such databases (Jimmy et al., 1994; Xuemei Han et al., 2008). To identify novel sequences and ORFs, these annotation databases were subsequently expanded with the inclusion of peptide sequences derived from genetically predicted coding regions. However, a number of additional factors, such as translation efficiency and post-transcriptional regulation, complicate the ability to accurately predict biologically relevant peptide products from transcriptional data alone (Schwanhäusser et al., 2011; Vogel and Marcotte, 2012). Additionally, events contributing to the multiplicity of proteoforms, including alternative splicing and PTMs, can be challenging–and at times impossible–to detect at the RNA level (Smith and Kelleher, 2013; Jian et al., 2014). One possible solution is to couple NGS with ultra-high-resolution MS to identify *de novo* peptides that may serve as promising biomarker candidates (Abecasis, 2010; Ning and Nesvizhskii, 2010; Gargis et al., 2012; Wang et al., 2012; Kamalakaran et al., 2013; Sheynkman et al., 2013; Chrystoja and Diamandis, 2014; Zhang et al., 2019). Disease-specific genomic variants can be identified from high-quality sequencing of disease-relevant tissue samples and used to build customized libraries for peptide biomarker identification via discovery proteomics (Figure 1). Recent success in both integrated proteogenomic analyses as well as variant protein detection is driving biomarker discovery and patient stratification in recent years.

## 2 Proteogenomics driving biomarker studies

### 2.1 Cancers

Strategies combining genomics and proteomics in the identification of cancer protein biomarkers have perhaps best demonstrated the utility of proteogenomics for biomarker discovery. The Cancer Genome Atlas program (TCGA) represents a rich resource for large-scale genomic data. TCGA comprises more than 30 cancer subtypes and provides data from both cancer and control tissue (Cancer, 2006; Tomczak et al., 2015). By integrating proteomics, the Clinical Proteomic Tumor Analysis Consortium (CPTAC) has sought to expand on this dataset, performing proteomic and PTM analysis on TCGA specimens. This effort has produced robust, multidimensional proteomic datasets of cancer tissue subtypes for groups seeking to conduct integrated proteogenomic analyses (Proteomics Cancer, 2007; Ellis et al., 2013a). Several studies have successfully demonstrated the utility of these datasets in uncovering candidate biomarkers (Rodriguez et al., 2021). For example, Chiou and colleagues successfully used these data to identify S100A9 and GRN as combinatorial biomarkers for early identification of hepatocellular carcinoma (HCC) from urine (Chiou and Lee, 2016). Moreoever, Gillete and colleagues leveraged the CPTAC database to perform proteogenomic characterization of lung adenocarcinoma (LUAD) and normal, adjacent tissue (Gillette et al., 2020). This analysis utilized not only proteomic and PTM data, but also whole-exome sequencing (WES), RNA-sequencing (RNAseq), and DNA methylation analysis, to identify mRNA and peptides derived from somatic mutations as biomarker candidates of LUAD driven by ALK-fusion where fusion proteins EML4-ALK with and HMBOX1-ALK were formed at transcriptome level (Table 1).

**TABLE 1:** Integrated proteogenomic analyses lead to cancer biomarker discovery.

Disease	Specimen	Brief summary	Ref
Cancer (Breast)	Patient tissue	Proteogenomics expression profiles used to determine drug resistance in breast cancer subtypes and understand drivers of oncogenic pathways	Lawrence et al. (2015)
Cancer (HCC)	Patient urine	Identification of HCC diagnostic biomarkers, proposing S100A9 and GRN as potential combinatorial biomarkers	Huang et al. (2015)
Cancer (Neuroblastoma, Colorectal)	Cultured cells	Mutant proteins released by extracellular vesicle subtypes elucidate the role of EVs in cancer progression and identify possible diagnostic biomarkers in easily-accessible biofluids	Keerthikumar et al. (2015)
Cancer (Breast)	Patient tissue (TCGA)	Proteomic and phospho-proteomic data combined with TCGA transcriptomic data to classify breast cancer subtypes and identify candidate drug targets	Mertins et al. (2016)
Healthy B-cells	Cultured Cells	Proteogenomic identification and analysis of MHC-I associated peptides (MAPs) from previously unidentified reading frames, revealing the potential for non-coding or “cryptic” MAPs as a source of tumor-specific antigens	Laumont et al. (2016)
Cancer (Prostate)	Patient tissue	Proteogenomic profiling, demonstrating the utility of mutliomics in the generation of novel prostate cancer subtypes; supports the adoption and expansion of research developing multimodal markers	Sinha et al. (2019)
Cancer (Breast)	Patient tissue (Oslo2, TCGA)	Study achieving both the recapitulation of the established PAM50 breast cancer subtypes, as well as further stratification-based proteogenomic profiles	Johansson et al. (2019)
Cancer (Endometrial)	Patient tissue (CPTAC)	A proteogenomic analysis with the notable inclusion of circRNA, acetylation contributes unique insights into the development of endometrial carcinoma and the consequences of specific mutational profiles and proposes novel endometrial carcinoma subtypes	Dou et al. (2020)
Cancer (Lung)	Patient tissue (CPTAC)	CPTAC study that identifies a number wild-type proteins and ALK-fusion products as potential biomarkers in LUAD and proposes a number of PTMs holding potential diagnostic value	Gillette et al. (2020)
Cancer (Lung)	Patient tissue	Study identifying demographic risk factors for early-onset LUAD, possible biomarkers for patient stratification, and druggable targets in early-stage LUAD.	Chen et al. (2020)
Cancer (Glial)	Patient tissue (SMC)	Study proposing classifications of previously-thought-to-be glioblastoma subtype, holding both prognostic value and the potential to inform personalized treatment	Oh et al. (2020)
Cancer (Brain)	Patient tissue	Study in which proteogenomic analysis integrating a number of pediatric brain tumor subtypes reveal common therapeutic vulnerabilities across subtypes	Petralia et al. (2020)
Cancer (Glial)	Patient tissue	Proteogenomic analysis revealing patient subtypes based on immune profiles, demonstrating a multidimensional strategy applicable for both further mechanistic investigation and patient stratification	Wang et al. (2021)
Cancer (Lung)	Patient tissue (CPTAC)	CPTAC study clustering analysis revealed both tumor subtypes and specific therapeutic vulnerabilities	Satpathy et al. (2021)
Cancer (Pancreatic)	Patient tissue (CPTAC)	Proteogenomic approach yielding a rich subset of biomarkers with potential for detection, diagnosis, and treatment	Cao et al. (2021)
Cancer (Breast)	Patient tissue (CPTAC)	Proteogenomic analyses unveiled *19q13.31–33* deletion as a marker associated with chemotherapy resistance	Anurag et al. (2022)

Tumor-specific somatic mutations are ideal targets for biomarker development. For example, targeted MS-based detection of mutant KRAS_p.G12V_ and KRAS_p.G12D_ proteins has proven to be a viable biomarker strategy in colorectal and pancreatic cancers (Wang et al., 2011). In addition to oncogenic mutations, tumors have also been found to contain up to 100 “passenger” mutations, many of which are translated into potentially targetable proteins (Reddy et al., 1982; Wood Laura et al., 2007; Stratton et al., 2009; Bignell et al., 2010; Bozic et al., 2010). Although many disease-associated mutations have been identified over the years, including *KRAS* (Demory Beckler et al., 2013), *P53* (Duffy et al., 2018), and *EGFR* (Awasthi et al., 2018), the vast heterogeneity of mutation sites not only poses a challenge to forming effective therapies, but also makes the possibility of creating antibodies for each mutation impractical (Leonardi et al., 2012). MS-based proteogenomics is often employed to discover mutant and novel peptides that occur downstream of tumor-specific mutations and hold promise as future biomarker candidates.

### 2.2 Neurodegenerative diseases

Similar to cancer, there is an increasing role for biomarkers of disease characterization and patient stratification in the field of neurodegeneration (DeKosky and Marek, 2003). Despite the fact that there has been limited success in identifying true plasma or cerebrospinal fluid (CSF) biomarkers of neurodegenerative disease thus far (Carlyle et al., 2018), there has been recent, promising progress in this field, assisted by proteogenomic strategies.

#### 2.2.1 Alzheimer’s disease

Using an integrative proteogenomic pipeline, Li and colleagues successfully identified 496 novel peptides in AD postmortem brain tissue. These identified peptides represent translational products of mutations and mis-splicing events that occur in AD and could serve as putative protein biomarkers (Li et al., 2016a). Applying a proteogenomic approach that was specifically designed to dissect alternative splicing events, Johnson et al. identified modules associated with AD cognitive decline using co-expression network analyses of postmortem brain samples. From these modules, the investigators then identified a number of differentially expressed, novel alternative splice variant proteins (Johnson et al., 2018).

Validation of biomarker candidates through large-scale studies of human samples is an essential component of developing clinical-grade biomarkers. To that end, high-throughput targeted MS-based approaches are often employed to validate findings discovered through companion shotgun proteomics approaches. For example, a targeted proteomics assay was recently used to identify APOE4-specific peptides in the plasma of AD patients (Simon et al., 2012). Expanding on the conventional identification of tau protein for clinical diagnosis of AD, multiple phospho-tau proteins were quantified using targeted proteomics of postmortem brain and CSF from AD patients (Barthelemy et al., 2019). Similarly, exon-specific 4R tau isoform-derived tryptic peptides were successfully quantified by targeted MS in the CSF of patients with Lewy body dementia (Barthelemy et al., 2016).

#### 2.2.2 Frontotemporal dementia and amyotrophic lateral sclerosis (FTD/ALS)

During the past two decades, several pathological mechanisms of FTD and ALS involving TDP-43, Tau, and SOD1 have been extensively described (Hedl et al., 2019). Mutations in *C9orf72*, *TDP-43*, *FUS*, and *VCP* have been found to be closely associated with FTD/ALS and represent promising biomarker candidates; however, there is still an absence of protein biomarkers for early disease detection. (Abramzon et al., 2020). Recently, an ultra-sensitive MS assay was used to successfully quantify C9ORF72 isoform levels in human brain tissue, demonstrating a significant decrease of the C9ORF72 long isoform in the brains of C9ORF72 mutation carriers (Viode et al., 2018). Additionally, TDP-43 pathology-related cryptic exon RNAs translated protein product have been observed in induced pluripotent stem cells derived neurons with TDP-43 deficiency as well as in CSF from FTD-ALS patients; this may represent a viable target for peptide-based biomarker development (Ling et al., 2015; Seddighi, 2023).

#### 2.2.3 Huntington’s disease

Huntington’s Disease (HD) is caused by a CAG repeat expansion, leading to accumulation and impaired clearance of mutant huntingtin protein. HD is currently diagnosed on the basis of a direct genetic test for CAG repeats, and performance on cognitive tests is the primary metric for disease progression (Yamamoto et al., 2000; Killoran et al., 2022). The need for an objective and sensitive biomarker for HD prognosis led to the identification of mutant huntingtin protein in CSF via an immunoprecipitation and flow-cytometry based assay (Southwell et al., 2015). A biomarker panel combining mutant and native proteins could aid in earlier diagnosis of the disease. Recent investigations have not only identified mutant huntingtin proteins in the mouse cortex using targeted MS approaches (Sap et al., 2021), but also demonstrated that combining mutant huntingtin protein and native markers (e.g., neurofilament light) can enable earlier HD detection and effective monitoring of disease progression and response to treatment (Rodrigues et al., 2020).

## 3 Translational value of proteogenomic biomarker strategies

### 3.1 Diagnosis and prognosis

To date, the most common application of biomarkers has been in the context of disease diagnosis. Monitoring the levels of native proteins has paved the way for accurate detection of breast cancer (Gam, 2012), colon cancer (Kuppusamy et al., 2017), pancreatic cancer (Duffy et al., 2010), and neurodegenerative diseases (Heywood et al., 2015). However, there is an emerging role for the implementation of mutant protein biomarkers in disease detection. Following the established role of *BRAF* mutations in cutaneous melanoma, which often results in the substitution of glutamic acid for valine at position 600 (*BRAF*_*V600E*_), this genetic signature and its protein products have garnered much attention as both a diagnostic and prognostic biomarker for melanoma (Capper et al., 2011; Ghossein et al., 2013; Long et al., 2013).

Biomarker panels have demonstrated utility in detecting disease with both specificity and sensitivity. In 2017, Cohen and colleagues presented a proteogenomic screening test for the detection of pancreatic ductal adenocarcinoma using a joint panel of four conventional protein biomarkers for cancer, combined with the presence of mutant *KRAS* circulating tumor DNA (ctDNA) from a blood draw. With 64% specificity, 99.5% sensitivity, and a demonstrated prognostic value for overall survival, this combinatorial strategy has considerable promise for earlier detection of pancreatic cancer (Cohen et al., 2017). A year later, this strategy was expanded further by CancerSEEK, implementing a panel of ctDNA, consisting of 61 amplicons spread across 16 genes, combined with 8 protein biomarkers. CancerSEEK allows for detection of breast, colorectal, esophageal, liver, lung, ovarian, pancreatic, and stomach cancers from a single blood sample with a specificity of 99% and a sensitivity between from 69%–98%, depending on the type of cancer (Cohen et al., 2018). The efforts from Cohen et al. highlight the potential of proteogenomic panels for a variety of diseases.

### 3.2 Patient stratification

In addition to diagnostic and prognostic applications, biomarkers enable patient stratification, allowing for informed and individualized treatment courses. The use of large-scale data to identify “treatable traits” in patients has been a topic of intense focus (König et al., 2017), as conventional classifications based on generalized markers have led to misclassification and ineffective treatment of clinically and pathologically heterogeneous disorders (Nevo et al., 2016). In an attempt to expand upon the five currently implemented breast-cancer subtypes derived from a set of 50 transcriptional signatures (i.e., PAM50 markers) (Parker et al., 2009), Johansson et al. utilized an integrated proteomics analysis on tumor tissue from patients representing each of the five PAM50 subtypes. (Johansson et al., 2019). In addition to identifying proteins derived from non-coding regions as candidate immunotherapeutic targets, network analyses succeeded in stratifying known patient classifications further, proposing previously unrecognized biomarkers and subclasses to guide therapeutic development.

Two studies in lung adenocarcinoma have also highlighted the potential of applying proteogenomics in patient stratification. Chen et al. revealed 5 mutational profiles previously unidentified in LUAD in an East Asian cohort (Chen et al., 2020). The group identified protein and genetic signatures in these subtypes strongly tied to age, gender, and *EGFR*-mutation status, contributing important considerations for the development of disease-modifying therapies. Furthermore, integrated analyses of multi-omics data from glioblastoma (GBM) samples unveiled new immune-based subtypes, expanding on previous classifications based only on transcriptomic and genomic data (Wang et al., 2017; Wang et al., 2021). Notably, the study subdivided glioblastoma into two distinct groups, allowing for future, more in-depth mechanistic studies to reveal therapeutic vulnerabilities in these newly discovered subclasses for precision medicine (Oh et al., 2020). Leveraging genomic, transcriptomic, and proteomic data together has provided rich resources for better patient stratification, as well as the identification of potential biomarker and therapeutic targets.

## 4 Biomarker discovery workflow using proteogenomics

### 4.1 Genomics generates variant databases for proteomics

**FIGURE 2:**
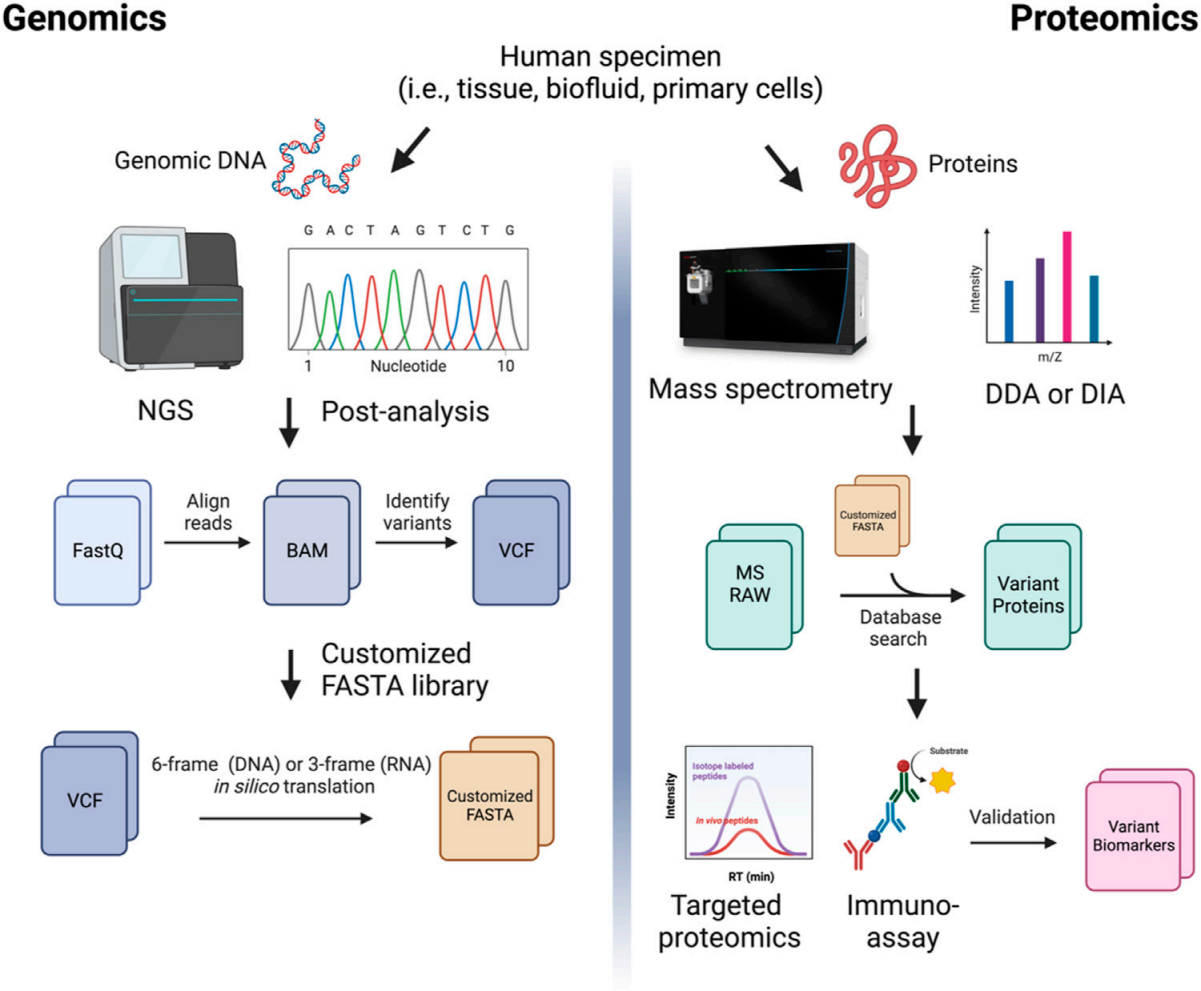
A comprehensive proteogenomic strategy in biomarker discovery. Genomic sequencing reads are aligned to the reference transcriptome to generate BAM files. Variants are called from aligned reads (i.e., Variant Call Format, VCFs). The VCF files are 6-frame (for DNA) or 3-frame (for RNA) translated to produce customized protein sequence (FASTA) files. Mass spectrometry (MS)-based protein sequencing using data-dependent acquisition (DDA) or data-independent acquisition (DIA) is performed. The MS raw files are searched against the custom library generated from genomic data. Identified variant biomarker candidates are validated using targeted proteomics or antibody-based immunoassays in large cohort studies.

Here, we propose a general MS-based proteogenomic workflow for the identification of variant protein markers in human biospecimens (Figure 2). The first step in creating customized databases capable of detecting variants in MS-based approaches is to identify disease-relevant genomic variants. Informatic tools for variant calling are widely available. The most common variants are SNV variants—commonly identified through tools such as Platypus (Rimmer et al., 2014) and Samtools (Li, 2011)—and splicing variants—which can be identified using MAJIQ (Vaquero-Garcia et al., 2016) and MapSplice (Wang et al., 2010), among other tools. Novel peptide products can be predicted from RNA-sequencing results via ECgene (Lee et al., 2006), FastDB (De La Grange et al., 2005), FANTOM3 (Carninci et al., 2005), or the ASTD (Koscielny et al., 2009). Novel protein sequences generated from *in silico* translation of the reference genome and/or transcriptome—e.g., via tools such as AGUSTUS (Stanke et al., 2006), GENEID (Parra et al., 2000) or EuGENE (Foissac et al., 2003)—allow for customized databases with the power to identify and validate proteins and peptides translated from antisense strands, non-coding genes, intergenic regions, and untranslated regions (UTRs) (Nesvizhskii, 2014). Once the RNA sequences of interest are identified, *in silico* translation tools, such as Transeq (CITE), Quilts (Ruggles et al., 2016), and GalaxyP (Sheynkman et al., 2014), can be used to predict the resulting amino acid sequence and build a custom peptide database. With this customized FASTA database, it is possible to perform searches of proteomics raw files for sequences of interest using MS search engines, such as PEAKS (Tran et al., 2019), Proteome Discoverer, and MaxQuant (Cox and Mann, 2008).

Integrated proteogenomic algorithms are also available for “one-stop” analyses, starting from variant calling to MS-spectra annotation (Table 2); however, some tools are not as popular as database search engines and have not been thoroughly validated. Beyond generating patient-specific databases, common mutations from existing databases (Table 3) can be introduced to native proteome databases. For example, Catalogue of Somatic Mutations in Cancer (COSMIC), containing somatic mutations from variety of cancer types, has been widely used for generating customized reference and identifying cancer-specific mutations (Zhu et al., 2018). Qi and colleagues utilized LNCipedia to predict lncRNAs regions and discovered lncRNA-coded neoantigens in lung adenocarcinoma (Qi et al., 2021). A key consideration in developing a proteogenomic database search strategy is the determination of an appropriate false-discovery rate (FDR). By increasing the database size through the integration of native plus variants proteome, the identified variant peptides are prone to high false positive rates from multiple comparisons. Therefore, additional targeted methods are required for validation.

**TABLE 2:** Informatic tools for creating customized protein sequence libraries using RNA-seq data.

Tool	Purpose	Link to tool	Ref
GalaxyP	Creates customized proteomic databases suitable for discovery proteomics using RNA-seq data	http://galaxyp.org	Sheynkman et al. (2014)
MiTPeptideDB	Bioinformatic workflow for detection of novel peptides from RNA-seq data, including filters for peptide detectability	http://bit.ly/MiTPeptideDB	Guruceaga et al. (2020)
Quilts	Integrates sample-specific genomic and transcriptomic data to predict peptides resulting from single nucleotide variants, splice variants, and fusion genes	http://fenyolab.org/tools/tools.html	Ruggles et al. (2016)
Proteoformer	Uses ribosome profiling data to create peptide product databases	http://www.biobix.be/proteoformer	Crappe et al. (2015)
JUMPg	Uses RNA-seq data to generate databases of DNA polymorphisms, mutations, and splice junctions, as well as six-frame protein fragments	https://github.com/gatechatl/JUMPg	Li et al. (2016b)
IPAW	Predicts peptide products across the full range of the tryptic peptidome, including pseudogenes, lncRNAs, short ORFs, alternative ORFs, N-terminal extensions, and intronic sequences, searches target and decoy databases, and provides an FDR-value for novel and variant peptides	https://github.com/lehtiolab/proteogenomics-analysis-workflow	Zhu et al. (2018)
PGA	Creates customized protein databases from RNA-seq data without reliance on a reference genome, searches tandem mass spec datasets, and identifies novel peptides	http://bioconductor.org/packages/3.8/bioc/html/PGA.html	Wen et al. (2016)
Peppy	Generates peptide and decoy databases from RNA-seq data, matches peptides to MS/MS spectra, and assigns confidence values to matches	http://geneffects.com/peppy	Risk et al. (2013)
Splicify	Combines RNA-seq and tandem mass spectrometry data to identify protein isoforms that arise from differential splicing	https://github.com/NKI-TGO/SPLICIFY	Komor et al. (2017)
FusionPro	Predicts translation products of fusion genes using a transcriptome-informed approach to identify fusion junction isoforms	https://bitbucket.org/chaeyeon/fusionpro	Kim et al. (2019)
PoGo	Peptide-to-genome mapping tool	https://www.sanger.ac.uk/tool/pogo/	Schlaffner et al. (2017)
PGx	Maps peptides onto genomic coordinates	https://github.com/FenyoLab/PGx	Askenazi et al. (2016)

**TABLE 3:** Databases of common genetic variants and MS data repositories.

Database	Purpose	Link to database	Ref
COSMIC	Catalogue of Somatic Mutations in Cancer	https://cancer.sanger.ac.uk/cosmic	Tate et al. (2019)
TCGA	Database of raw and processed genome sequencing data for over 30 human tumors	https://gdc.cancer.gov/	Hoadley et al. (2018)
CPTAC	Mass spectrometry-based proteomic dataset for selected breast, colon, and ovarian tumors from TCGA	https://gdc.cancer.gov/about-gdc/contributed-genomic-data-cancer-research/clinical-proteomic-tumor-analysis-consortium-cptac	Ellis et al. (2013b)
Human Protein Atlas	Database of human proteins in cells, tissues, and organs uisng multi-omics appoarches and system biology	https://www.proteinatlas.org/	Uhlen et al. (2015)
ProteomeXchange	Regularly updated repository of over 8,000 human (including cell lines) MS/MS proteomics and SRM datasets	http://www.proteomexchange.org/	Vizcaino et al. (2014)
LNCipedia	Public database for long non-coding RNA (lncRNA) sequence and annotation	https://lncipedia.org/	Volders et al. (2019)
PeptideAtlas	Compendium of results from >150,000 MS runs processed through the Trans Proteomic Pipeline	http://www.peptideatlas.org/builds/human/	Desiere et al. (2006)
DEPOD	Database of human phosphatases, their protein and non-protein substrates, and dephosphorylation sites	http://www.depod.org	Duan et al. (2015)
ActiveDriverDB	Proteogenomic database of PTM-associated mutations in human disease	https://www.ActiveDriverDB.org	Krassowski et al. (2018)

### 4.2 Identification of variant protein biomarkers

Similar to NGS approaches, MS-based proteomics has rapidly advanced throughout the past two decades. Performing total RNA-seq in biofluids has proven to be technically challenging (Everaert et al., 2019). Given the low quantity and quality of RNA in biofluids, most biomarker studies focus on circulating DNA and small RNAs (Buschmann et al., 2016; Vo et al., 2019). Therefore, protein biomarkers have become the most common clinical markers in body fluids. To increase proteome coverage, various approaches have been adopted. These include a) offline fractionation to reduce sample complexity; b) high-abundant protein depletion to remove housekeeping proteins in biofluids; c) enrichment for tissue-derived extracellular vehicles (EVs) (Fiandaca et al., 2015; Mustapic et al., 2017; Heath et al., 2018); d) nanoparticle-based enrichment of low-abundant proteins and co-depletion of high-abundant proteins (Kim et al., 2018; Tiambeng et al., 2020); and e) use of multiple proteases to detect peptides not typically generated by standard trypsin cleavage (Giansanti et al., 2016).

For data acquisition in discovery proteomics, data-dependent acquisition (DDA) and data-independent acquisition (DIA) are commonly used in MS. Previous studies demonstrated that DDA and DIA acquire different groups of peptides; this could extend the pool of total peptide identification and protein coverage (Reilly et al., 2021). DDA typically generates less complex, but more specific, MS2 spectra of selected peptides; however, only the most abundant peptide precursors are selected. On the other hand, DIA is a more inclusive approach to fragment all peptide precursors, including low-abundant ones. Although DDA has been more widely applied in biomarker studies, DIA has gained traction more recently for its applications in identifying low-abundant peptides (Guo et al., 2015; Latonen et al., 2018). The increased scan speed of high-resolution MS allows DIA to use narrower isolation windows and cover a broader m/z range (e.g., 400–1,000). DIA generally provides higher confident peptides due to the longer MS2 injection time, which allows for high-resolution MS2 spectra. Database search of DIA data typically requires a spectral library generated from the respective DDA MS run; notably, recent studies demonstrate the direct application of DIA data using a protein sequence library where “pseudo-spectra” and predicted retention times of each precursor ion is generated by search engines, such as DIA-Umpire (Tsou et al., 2015), Spectronaut, and DIA-NN (Demichev et al., 2020). Emerging evidence shows DIA is the next-generation data acquisition approach for label-free proteomics.

Targeted proteomic analyses are commonly employed to validate mutant peptides discovered through DIA/DDA shotgun proteomics and to generate high-throughput MS-based assays for clinical use. Targeted approaches, including multiple reaction monitoring (MRM) and parallel reaction monitoring (PRM), align select or all MS2 transitions and retention times of *in vivo* peptides and their “heavy isotope” synthetic counterparts that serve as internal standards. Typically, a list of m/z ratio of the precursor ions and their daughter ions is built into the MS instrumentation method to selectively monitor targets. Furthermore, DIA is a “semi-targeted” approach, as the MS2 transitions that are used for qualification can also be visualized as PRM-like spectra in Skyline (MacLean et al., 2010) and SpectroDive. Many proof-of-concept studies have utilized targeted methods to validate variant peptides, as the “gold standard,” ultra-sensitive approach. The biomarker specificity of validated peptides should also be demonstrated in large-scale cohorts containing disease and healthy control samples. If the variant peptides are validated as specific biomarkers, scalable MS-based MRM assays can be developed to rapidly detect such biomarkers in patient samples for point-of-care diagnosis and disease subtype stratification.

## 5 Perspective

Combining NGS and MS-based proteomics represents a powerful strategy for both biomarker discovery and investigation of fundamental biology. However, obtaining sufficient high-quality RNA-seq reads can be challenged by the integrity and quantity of available biospecimens. Furthermore, short-read RNA-seq could easily miss mutation sites and mis-splicing events; therefore, long-read RNA-seq has emerged as a complementary approach, despite its shallower sequencing depth. Although proteome coverage has significantly improved in recent years, low-abundant proteins may still be difficult to identify with current tools. Many approaches have been applied to increase protein coverage, but they are generally time-consuming and increase intra-sample variation. Clinical assays must be quick, robust, and highly reproducible. Therefore, MS instrumentation and proteomic sample preparation need further improvement to boost sensitivity and specificity. *De novo* proteins could also be structurally unstable and degraded by proteases and peptidases within the lysosome and endosome, thereby evading detection. Overall, despite these challenges, sequence-centric approaches, combined with state-of-the-art mass spectrometry, contribute to the evolving role of proteogenomics in biomedical research and precision-medicine based initiatives in cancer, neurodegeneration, and beyond.
